# Significance of CXCL1 chemokine in the development of tau pathology

**DOI:** 10.1002/alz70856_103793

**Published:** 2025-12-26

**Authors:** Agnieszka Kulczynska‐Przybik, Daria Krawczuk, Renata Borawska, Izabela Winkel, Barbara Mroczko

**Affiliations:** ^1^ Department of Neurodegeneration Diagnostics, Medical University of Bialystok, Bialystok, Poland; ^2^ Dementia Disorders Centre, Medical University of Wroclaw, Scinawa, Poland

## Abstract

**Background:**

Multiple investigations on the pathological mechanisms of Tau protein in the course of Alzheimer's disease (AD) have been carried out, however, the exact processes are still unclear. One of the crucial factors contributing to the development of AD seems to be chemokine CXCL1 produced by neurons and cells of the immunological system in the brain. It was reported that monocytes from AD patients express significantly elevated levels of CXCL1, additionally, this chemokine is involved in monocyte migration from the blood to the brain in AD patients. Furthermore, some evidence from studies performed on neuronal lines and animal models indicates that CXCL1 contributes to caspase‐3‐dependent tau cleavage, which may result in abnormal distribution of the tau protein. In pathological conditions, CXCL1 activates the CXCR2 receptor on neurons, which causes the activation of pathways and leads to the hyperphosphorylation of tau. Therefore, we aimed to assess the CXCL1 levels in CSF patients with AD and cognitively normal subjects and compared them with CSF and plasma indicators of tau protein pathology.

**Method:**

The concentrations of CXCL1 as well as neurochemical dementia biomarkers were measured in cerebrospinal fluid and plasma patients with AD and patients without cognitive decline by multiplexing, SIMOA, and enzyme‐linked immunosorbent techniques.

**Result:**

Significantly higher CSF concentrations of CXCL1 were found in AD patients compared to the subjects without cognitive decline. Furthermore, in the group of patients with AD, the levels of CXCL1 significantly correlated with CSF pTau181 protein. Similarly, a significant association between CSF CXCL1 and Tau plasma protein was observed.

**Conclusion:**

Our findings indicate that CXCL1 may play a role in the pathogenesis of AD, particularly in the intensified mechanism of Tau protein.

